# Immunogenic Cell Death by the Novel Topoisomerase I Inhibitor TLC388 Enhances the Therapeutic Efficacy of Radiotherapy

**DOI:** 10.3390/cancers13061218

**Published:** 2021-03-11

**Authors:** Kevin Chih-Yang Huang, Shu-Fen Chiang, Pei-Chen Yang, Tao-Wei Ke, Tsung-Wei Chen, Ching-Han Hu, Yi-Wen Huang, Hsin-Yu Chang, William Tzu-Liang Chen, K. S. Clifford Chao

**Affiliations:** 1 Department of Biomedical Imaging and Radiological Science, China Medical University, Taichung 40402, Taiwan; T96752@mail.cmuh.org.tw; 2 Translation Research Core, China Medical University Hospital, China Medical University, Taichung 40402, Taiwan; 3 Lab of Precision Medicine, Feng-Yuan Hospital, Ministry of Health and Welfare, Taichung 42055, Taiwan; A6263@mail.cmuh.org.tw; 4 Cancer Center, China Medical University Hospital, China Medical University, Taichung 40402, Taiwan; u9810029@cmu.edu.tw (P.-C.Y.); alice6406@hotmail.com (C.-H.H.); lucy6430@gmail.com (Y.-W.H.); che821115@gmail.com (H.-Y.C.); 5 Department of Colorectal Surgery, China Medical University Hospital, China Medical University, Taichung 40402, Taiwan; ketaowei@mail.cmu.edu.tw; 6 School of Chinese Medicine & Graduate Institute of Chinese Medicine, China Medical University, Taichung 40402, Taiwan; 7 Department of Pathology, Asia University Hospital, Asia University, Taichung 41354, Taiwan; runrunrungo@yahoo.com.tw; 8 Graduate Institute of Biomedical Science, China Medical University, Taichung 40402, Taiwan; 9 Department of Surgery, School of Medicine, China Medical University, Taichung 40402, Taiwan; 10 Department of Colorectal Surgery, China Medical University HsinChu Hospital, China Medical University, HsinChu 302, Taiwan; 11 Department of Radiotherapy, School of Medicine, China Medical University, Taichung 40402, Taiwan

**Keywords:** locally advanced rectal cancer, neoadjuvant chemoradiotherapy, topoisomerase I inhibitor, lipotecan, anticancer immunity

## Abstract

**Simple Summary:**

This study aims to evaluate the induction of immunogenic cell death (ICD) for anticancer immunity by the novel topoisomerase I inhibitor lipotecan. These results show that lipotecan can remarkably elicit ICD and increase tumor immunogenicity, which promotes the therapeutic efficacy of radiotherapy compared to conventional chemoradiotherapy in vivo. These results provide potential therapeutic strategies to improve the efficacy of chemoradiotherapy in colorectal cancer (CRC), which may increase the local control rate and decrease tumor relapse in locally advanced rectal cancer (LARC) patients who receive preoperative chemoradiotherapy.

**Abstract:**

Rectal cancer accounts for 30–40% of colorectal cancer (CRC) and is the most common cancer-related death worldwide. The preoperative neoadjuvant chemoradiotherapy (neoCRT) regimen is the main therapeutic strategy for patients with locally advanced rectal cancer (LARC) to control tumor growth and reduce distant metastasis. However, 30–40% of patients achieve a partial response to neoCRT and suffer from unnecessary drug toxicity side effects and a risk of distant metastasis. In our study, we found that the novel topoisomerase I inhibitor lipotecan (TLC388) can elicit immunogenic cell death (ICD) to release damage-associated molecular patterns (DAMPs), including HMGB1, ANXA1, and CRT exposure. Lipotecan thereby increases cancer immunogenicity and triggers an antitumor immune response to attract immune cell infiltration within the tumor microenvironment (TME) in vitro and in vivo. Taken together, these results show that lipotecan can remodel the tumor microenvironment to provoke anticancer immune responses, which can provide potential clinical benefits to the therapeutic efficacy of neoCRT in LARC patients.

## 1. Introduction

Rectal cancer accounts for 30–40% of colorectal cancer (CRC) cases [1,2]. Locally advanced rectal cancer (LARC), comprising T3-T4 tumors and/or locoregional lymph nodes metastasis, is difficult to cure. Preoperative neoadjuvant chemoradiotherapy (neoCRT) is currently considered the standard treatment for patients with LARC [3,4], containing a noncytotoxic radiosensitizing dose of fluoropyrimidine such as capecitabine and 5-fluorouracil (5-Fu) with fractionated radiotherapy (45–50 Gy in 25–28 fractions), followed by resection of the residual tumor tissue [5]. This multimodality treatment improved local control rates and resulted in complete tumor regression in 15–20% of patients, but 30–40% of patients still experienced distant metastasis within five years [6,7]. The five-year survival in patients with LARC is 45–75%, with recurrences occurring in 5–15% of patients [8,9]. The addition of postoperative adjuvant chemotherapy has no improvement in this setting [10]. Therefore, efforts have been made to improve the therapeutic efficacy of neoCRT regimens and LARC outcomes in clinical trials by the use of neoadjuvant chemotherapy (neoCT) prior to or immediately following radiotherapy, such as oxaliplatin and irinotecan [11,12,13,14,15]. However, the results of randomized trials have not been favorable in these combinations with increased acute toxicities [16], suggesting that the addition of other anticancer agents is still urgent.

Within all chemotherapeutic drugs used for cancer treatments, inhibitors of topoisomerases I and II represent important and intensively studied drugs, and these compounds can also manifest immune-enhancing effects because of extensive genomic damage [17,18]. The augmentation of tumor antigens by topoisomerase inhibitors greatly allows the T cell recognition of target cells. Topotecan (TPT) has been reported to trigger the secretion of damage-associated molecular pattern molecules (DAMPs) for the activation of DCs [19]. TPT is a camptothecin analog that inhibits topoisomerase I and triggers DNA double-strand breaks for cell death. TPT inhibits tumor growth by promoting DC maturation and CD8+ T cell activation in tumor-bearing mice. Notably, TPT treatment triggers the secretion of exosomes containing immunostimulatory DNA, which is taken up by DCs and activates a STING-dependent pathway [19]. Thus, these findings suggest that TPT treatment has the potential to act as an adjuvant to elicit antitumor immunity. The immunostimulatory activity of chemotherapeutic drugs has been linked to killing cancer cells accompanied by the activation of autophagy, endoplasmic reticulum (ER) stress, and type I IFN signaling for the anticancer immune response. The anticancer immune response of ICD is contributed by the release of damage-associated molecular pattern molecules (DAMPs) by dying cancer cells and stressed cancer cells, such as high-mobility group box 1 (HMGB1), heat shock protein 70 (Hsp70), ATP, annexin A1 (ANXA1), and calreticulin (CRT). These DAMPs play critical roles in shaping adaptive anticancer immune responses through the activation of immune cells such as dendritic cells (DCs), which is followed by tumor antigen processing and presentation to CD4^+^ and CD8^+^ T cells [20,21]. Therefore, the infiltration of tumor-infiltrating lymphocytes (TILs) is associated with the antitumor immune response within the tumor microenvironment (TME) [22,23,24,25,26]. High infiltration of T cells correlates with improved relapse-free and overall survival in patients with CRC [27,28,29,30].

Lipotecan (TLC388, Taiwan Liposome Company, Ltd., Taipei, Taiwan) is a novel camptothecin targeting topoisomerase I and is being developed to increase its antitumor potency. Lipotecan has demonstrated its anticancer ability in several human cancer cell lines and xenograft animal models, including lung, breast, colon, pancreas, liver, prostate, and ovarian cancers [31,32]. Lipotecan has been shown to enhance DNA double-strand breaks (DSBs) and inhibit DNA repair, which functions as a chemo- and radiosensitizing drug [31]. Moreover, lipotecan overcomes the intrinsic instability of the lactone E-ring, which is the common feature of CPT derivatives that reduce its potency and increase its toxicity on normal tissue [31]. Lipotecan is well tolerated and safe without cumulative toxicity and prolongs stable diseases in patients with metastatic chemotherapy-refractory solid tumors [32]. Previous studies showed that the common colorectal cancer irinotecan only enhanced HMGB release in vitro, suggesting its inefficiency in the induction of ICD [33]. Moreover, TPT did not induce conventional ICD, but it stimulated STING-dependent IFN-β production for DC activation [19]. Therefore, these two camptothecin derivatives were not immunogenic chemotherapeutic drugs, suggesting the improvement of anticancer agents to induce anticancer immunity is necessary. In our study, we found that lipotecan has significant cytotoxicity ability compared to topoisomerase I inhibitors topotecan and irinotecan. Moreover, lipotecan triggers ER stress and the release of DAMPs, such as CRT exposure and the release of HMGB1 and ANXA1. Lipotecan also enhances cancer immunogenicity. Compared to the 5-Fu-based CRT, the lipotecan-based CRT has a similar effect on tumor inhibition in vivo. However, lipotecan-based CRT results in better complete regression, high caspase-3 activation, and high immune cell infiltration. Taken together, these results show that the novel topoisomerase I inhibitor might increase the therapeutic efficacy of radiotherapy by provoking an anticancer immune response.

## 2. Methods and Materials

### 2.1. Cell Culture

Two colorectal cancer cell lines, SW480 (ATCC: CCL-22) and CT26 (ATCC: CRL-263), were obtained from the American Type Culture Collection (ATCC, Manassas, VA, USA). The cells were cultured and maintained in complete RPMI 1640 medium supplemented with 10% fetal bovine serum (Thermo Fisher Scientific, South San Francisco, CA, USA), 100 U/mL penicillin, and 100 mg/mL streptomycin at 37 °C in a humidified, 5% CO_2_ atmosphere.

On the day before treatment, SW480 and CT26 cells were seeded onto a 6 cm dish at ~80% confluence, and then cells were harvested for Western blot and flow cytometry analysis at the indicated times.

### 2.2. Antibodies and Reagents

The antibodies used in this study were as follows: anti-β-actin (sc-8432, Santa Cruz, Santa Cruz, CA, USA), anti-HMGB1 (No. 3935, Cell Signaling Technology, Danvers, MA, USA), anti-ANXA1 (No. 3299, Cell Signaling Technology), anti-cleaved caspase-3 (No. 9661, Cell Signaling Technology), anti-p-eIF2α (No. 3398, Cell Signaling Technology), anti-eIF2α (No. 9722, Cell Signaling Technology), anti-CRT (ab2907, Abcam, Cambridge, UK), anti-HSP70 (No. 4872, Cell Signaling Technology), and-HSP90 (No. 4875, Cell Signaling Technology) and HRP-conjugated anti-mouse and rabbit IgG secondary antibodies (Santa Cruz).

### 2.3. Western Blot Analysis

To evaluate the ecto-HMGB1 and ecto-ANXA1 in the conditioned medium (CM), the cells were washed and incubated with a serum-free medium for 2 h when subconfluent. The medium was discarded, and the cells were incubated with a serum-free medium containing the indicated concentration and time of TLC. After treatment, the CM was harvested and centrifuged to remove debris and filtered through a 0.22 μm filter. The CM was concentrated by ultrafiltration using a Microcon filter (10,000-Da cutoff; Millipore, Bedford, MA, USA). Either the total lysates (30 μg) or secreted proteins (10 μg) were separated via 6–12% sodium dodecyl sulfate polyacrylamide gel electrophoresis (SDS-PAGE) and transferred onto a PVDF membrane (GE Healthcare, Amersham, UK).

Briefly, the transferred membranes were then blocked with 5% nonfat milk and probed with the indicated antibodies overnight at 4 °C, probed with HRP-conjugated secondary antibodies for 2 h at room temperature, and incubated with Immobilon Western Chemiluminescent HRP Substrate (Millipore, MA, USA). The digital images of the Western blot were acquired using an ImageQuant^TM^ LAS4000 digital imaging system (GE Healthcare, CA, USA). If necessary, the blot was stripped using Restore Western blot Stripping Buffer (Thermo Fisher Scientific, CA, USA) and incubated with the other antibodies. The image quantification was evaluated using ImageJ software (NIH, Bethesda, MD, USA).

### 2.4. Evaluation of the Immunogenic TME Induced by Radiotherapy and Chemotherapeutic Drugs

BALB/c mice (female, 4 weeks old) were maintained according to the institutional guidelines approved by the China Medical University Institutional Animal Care and Use Committee (Protocol No. CMUIACUC-2019-293). Briefly, 100 μL Matrigel (Corning, Union City, CA, USA) containing fresh 5 × 10^5^ CT26 cells was inoculated subcutaneously into the right leg of each mouse. After 7 days when the tumor volume reached 70–100 mm^3^, the mice were randomly assigned into different groups and administered with 5-Fu (50 mg/kg/mouse, intraperitoneal injection) and lipotecan (5 mg/kg/mouse, intraperitoneal injection) for three days consecutive with a one-day interval for 9 days (Days 7, 8, 9, 11, 12, 13, 15, 16, and 17). For radiotherapy, mice were anesthetized with 300 μL PBS with ketamine (140 mg/kg) and xylazine (3 mg/kg) by intraperitoneal injection before irradiation. Before radiotherapy, the dosimetry data on the irradiation square (8.5 cm × 8.5 cm, depth 5 cm) were collected to validate the dosage of irradiation (293.2 ± 4.4 cGy/300 MU). The local tumors were then received 3 × 5 Gy fractionated radiotherapy (6 MV X-ray with 400 MU/min, TrueBeam, Varian) on Days 10, 14, and 18. Following complete anesthesia, the right leg was placed in the square irradiation field (6 cm × 6 cm), and the mouse’s body was kept away from the leg. Radiation was delivered to the irradiation field with the center height of the tumor according to the X-ray beam collimator. The half-beam block was used to protect vital organs, and a 1.5 cm transparent tissue-equivalent bolus was used to cover the irradiated site for an even distribution of irradiation throughout the tumor. The dose was calibrated using a Radcal ion chamber (Monrovia, CA, USA). The tumor volume was measured every 3 days throughout the study. The longest and shortest diameters (L and W, respectively) of the tumors were measured using Vernier calipers (Sata, Shanghai, China) every 3 days, and tumor volume (V) was calculated by the formula: V = (L × W^2^)/2. The mice were sacrificed at the termination of the experiments, and the tumor tissues were collected for lysis, subjected to immunoblotting analysis, and stained by immunohistochemistry.

### 2.5. Detection of the Surface CRT, MHC Class I, and CD80 Levels on Tumor Cells

After treatment with indicated chemotherapeutic drugs for 24 h, SW480 and CT26 were isolated with dissociation buffer (Thermo Fisher Scientific, CA, USA) and then blocked with 5% BSA for 15–20 min. These cells were then stained with a PE-conjugated antihuman CD80 antibody (Clone 2D10, BioLegend, San Diego, CA, USA), a PE-conjugated antimouse CD80 (Clone 16-10A1, BioLegend), and an Alexa 488-conjugated anti-MHC class I antibody (clone 2G5, Novus Biologicals, Centennial, CO, USA). The surface markers were examined by a BD LSR-II flow cytometer (BD Biosciences, Mountain View, CA, USA), and data were further analyzed using FlowJo software (TreeStar, Ashland, OR, USA).

### 2.6. Assessment of Cell Growth and Apoptosis

The cell growth was assessed using a CCK-8 assay. Caspase-3 activity was assessed using a caspase-3 activity kit (K106, Biovision). For the evaluation of cell death in animal specimens, the terminal deoxynucleotidyl transferase (TdT)-mediated dUTP-biotin nick end labeling (TUNEL) kit (Roche, South San Francisco, CA, USA) was used following the manufacturer’s manual. To evaluate the TUNEL^+^ cells, the tumor tissue was reviewed at 20× magnification. Three fields were included to calculate the average number of TUNEL^+^ cells/100 cells.

### 2.7. Immunohistochemistry

The antibodies used in this study were as follows: antimouse CD3 (ab16669, Abcam), antimouse CD8a (ab217344, Abcam), and antimouse HMGB1 (ab79823, Abcam). Tissue slides (3 µm thickness) were stained with the HRP-conjugated avidin–biotin complex (ABC) from the Vectastain Elite ABC Kit (Vector Laboratories, Burlingame, CA, USA) and DAB chromogen (Vector Laboratories) and counterstained with hematoxylin. Staining for CD3 and CD8a was positive when detected in tumor-infiltrating lymphocytes (TILs) and was evaluated using a microscope (OLYMPUS BX53, Tokyo, Japan). To evaluate the infiltrating density of TILs, the central region of tumor tissue was reviewed at 40× magnification, and the amount of CD3 and CD8a^+^ TILs within the tumor bed was counted. The average number of CD8^+^ TILs in five high-power fields was included to examine the number of immune cells per square millimeter (No. of TILs/mm^2^) [34,35,36].

### 2.8. Enzyme-Linked Immunosorbent Assay (ELISA)

To detect the level of HMGB1, the conditioned medium was collected after treatment, centrifuged to remove debris, and analyzed by LEGEND MAX™ human HMGB1 (Biolegend, CA, USA) and mouse hmgb1 Kit (Elabscience, Houston, TX, USA) according to the manufacturer’s manual.

### 2.9. Statistical Analysis

Between-group comparisons were performed using an unpaired *t* test and ordinary one-way ANOVA (including Dunnett’s and Tukey’s multiple comparison test), and the two-sided *p*-value was reported for all tests by GraphPad Prism 7 statistical software (GraphPad Software, San Diego, CA, USA). * *p* < 0.05, ** *p* < 0.01 and *** *p* < 0.001 were the significant levels in this study.

## 3. Results

### 3.1. The Novel Chemotherapeutic Drug Lipotecan Can Elicit Surface Exposure of Calreticulin via Endoplasmic Reticulum Stress

**Figure 1:**
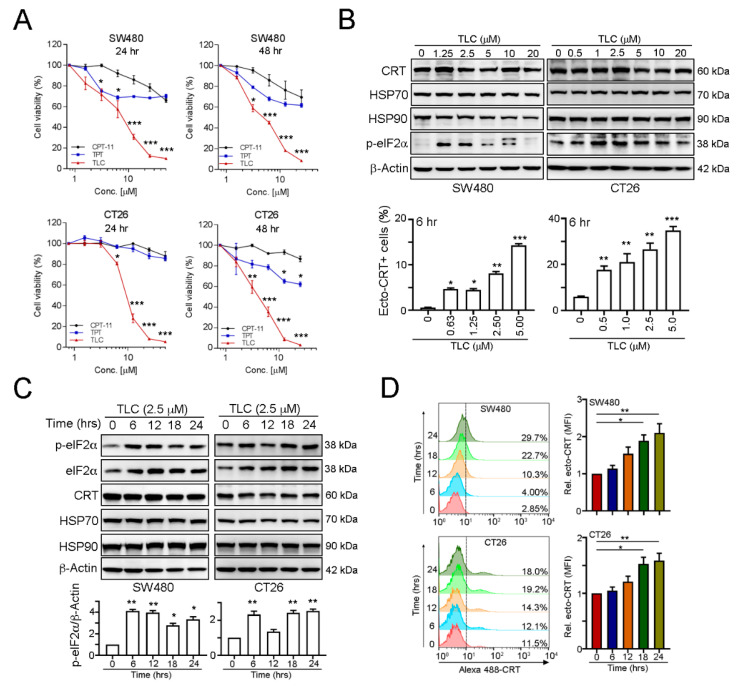
Lipotecan (TLC) triggered a decrease in cell viability and promoted endoplasmic reticulum (ER) stress. (**A**) SW480 and CT26 cells were treated with diverse concentrations of TLC for 24 and 48 h. Cell viability was examined by CCK-8 assay (*n* = 3). * *p* < 0.05, ** *p* < 0.01 and *** *p* < 0.001. (**B**) SW480 and CT26 cells were treated with diverse concentrations of TLC for 24 h and examined by immunoblotting. The level of surface CRT was analyzed by flow cytometry. Quantification of these results is shown (*n* = 3). * *p* < 0.05, ** *p* < 0.01 and *** *p* < 0.001. (**C**) SW480 and CT26 cells were treated with TLC for different time periods and examined by immunoblotting. Quantification of these results is shown (*n* = 3). * *p* < 0.05 and ** *p* < 0.01. (**D**) SW480 and CT26 cells were treated with TLC for different time periods and examined by flow cytometry. Quantification of these results is shown (means ± S.D.s., *n* = 3). * *p* < 0.05 and ** *p* < 0.01. More details of western blot, please view at Figures S1 and S2.

To evaluate whether lipotecan can elicit immunogenic cell death via ER stress, we first examined the cytotoxic activity of lipotecan on colorectal cancer cells. Compared to other topoisomerase I inhibitors, topotecan (TPT) and irinotecan (CPT-11), the cytotoxic ability of lipotecan was profound in SW480 and CT26 cells at 24 and 48 h (Figure 1A). Moreover, lipotecan treatment induced phosphorylation of the ER stress marker eIF2α and surface exposure to calreticulin (CRT) in a dose-dependent manner (Figure 1B). A low dose of lipotecan remarkably triggered eIF2α phosphorylation and ecto-CRT exposure in both SW480 and CT26 cells (Figure 1B). Furthermore, lipotecan quickly elicited eIF2α phosphorylation after 6 h of treatment in SW480 and CT26 cancer cells (Figure 1C). Surface exposure of CRT was significantly observed at 18 h after lipotecan administration in SW480 and CT26 cancer cells (Figure 1D). Taken together, these results suggested that lipotecan can not only directly damage cancer cells but also potentially trigger ER stress for CRT exposure, provoking anticancer immunity.

### 3.2. Lipotecan Remarkably Induces Immunogenic Cell Death (ICD) to Release HMGB1 and ANXA1 and Increase Cancer Immunogenicity

**Figure 2:**
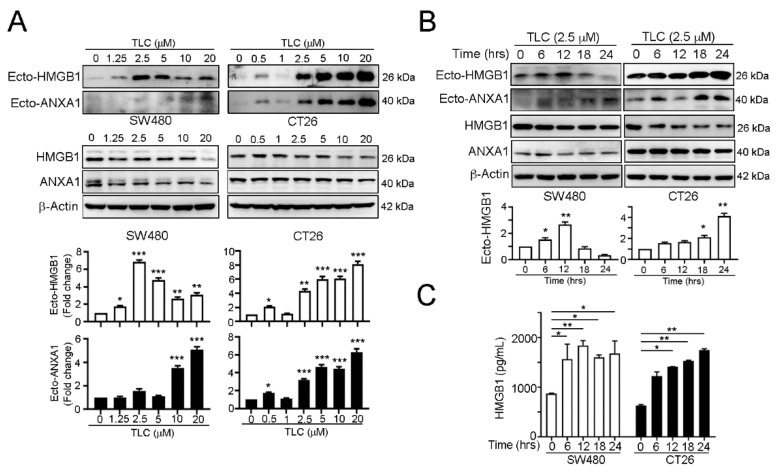
Lipotecan triggered HMGB1 and ANXA1 release. (**A**) SW480 and CT26 cells were treated with diverse concentrations for 24 h and examined by immunoblotting. The conditioned medium was concentrated and analyzed by immunoblotting. Quantification of these results is shown (*n* = 3). * *p* < 0.05, ** *p* < 0.01 and *** *p* < 0.001. (**B**) SW480 and CT26 cells were treated with 2.5 μM TLC for 6, 12, 18 and 24 h. The treated cells were harvested for immunoblotting. The quantification analysis is shown below (*n* = 3, * *p* < 0.05 and ** *p* < 0.01). (**C**) The conditioned medium from SW480 and CT26 cells that treated with 2.5 μM TLC for 6, 12, 18, and 24 h was harvested for HMGB1 ELISA (*n* = 3, means ± S.D.s., * *p* < 0.05 and ** *p* < 0.01). More details of western blot, please view at Figures S3 and S4.

To evaluate whether lipotecan induced immunogenic cell death (ICD), we detected two significant proteins, HMGB1 and ANXA1, which have been demonstrated to be damage-associated molecular patterns (DAMPs) for dendritic cell maturation via Toll-like receptor 4 (TLR4) and formyl peptide receptor 1 (FPR1) [34,37,38,39]. Secreted HMGB1 (ecto-HMGB1) and ANXA1 (ecto-ANXA1) were significantly detected at low doses of lipotecan in SW480 and CT26 cancer cell lines (Figure 2A). Furthermore, the secretion of HMGB1 and ANXA1 was time-dependent (Figure 2B,C), suggesting that lipotecan has the potential to elicit ICD, which profoundly promotes anticancer immunity.

**Figure 3:**
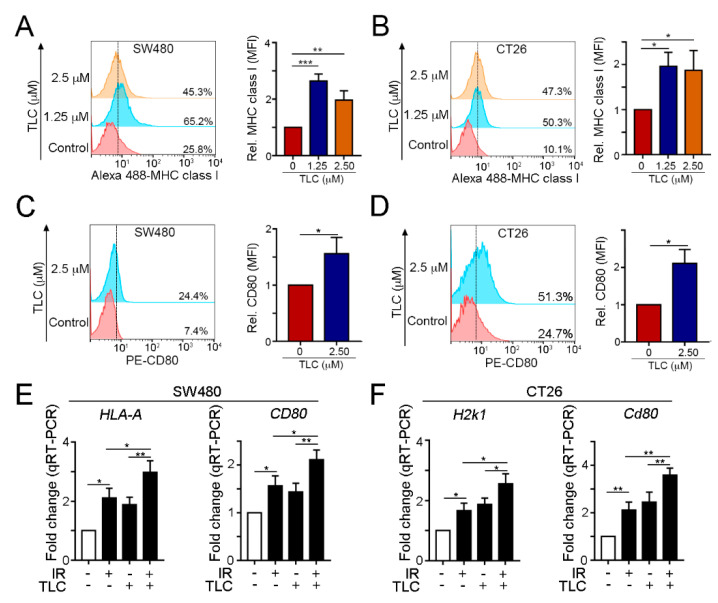
Lipotecan treatment triggered the immunogenicity of cancer cells. (**A**) SW480 cells were incubated with TLC for 24 h. The treated cells were harvested to examine the level of surface MHC class I was by flow cytometry. Quantification of these results is shown right (*n =* 3, ** *p* < 0.01 and *** *p* < 0.001). (**B**) CT26 cells were treated with TLC for 24 h, and the level of surface MHC class I was examined by flow cytometry. Quantification of these results is shown right (*n =* 3, * *p* < 0.05). (**C**) SW480 cells were treated with 2.5 μM TLC for 24 h, and the level of surface CD80 was examined by flow cytometry (*n* = 3, * *p* < 0.05.) (**D**) CT26 cells were treated with TLC for 24 h, and the level of surface CD80 was examined by flow cytometry. Quantification of these results is shown (*n* = 3). * *p* < 0.05. (**E**) SW480 cells were treated with TLC (2.5 μM) and IR (5 Gy) for 24 h, and the mRNA levels of HLA-A and CD80 were examined by qRT-PCR (*n =* 3). * *p* < 0.05 and ** *p* < 0.01, ordinary one-way ANOVA (Tukey’s multiple comparisons test). (**F**) CT26 cells were treated with TLC (2.5 μM) and IR (5 Gy) for 24 h, and the mRNA levels of *H2k1* and *Cd80* were examined by qRT-PCR (*n =* 3). * *p* < 0.05 and ** *p* < 0.01, ordinary one-way ANOVA (Tukey’s multiple comparisons test). These data were obtained from three independent experiments, and the values represent the means ± S.D.s.

To further investigate the effects of lipotecan on cancer immunogenicity, we analyzed the levels of MHC class I and CD80 by flow cytometry. We found that lipotecan significantly upregulated the levels of the antigen presentation markers MHC class I (Figure 3A,B) and CD80 (Figure 3C,D) in human and mouse colon cancer cell lines. The mRNA levels of the antigen presentation markers *HLA-A* (human MHC class I gene) and *CD80* in SW480 cells and *H2K1* (mouse MHC class I gene) and *Cd80* in CT26 cells were also increased after lipotecan treatment (Figure 3E,F). The cancer immunogenicity on cancer cells was remarkably increased following irradiation and lipotecan treatment (Figure 3E,F). These results showed that lipotecan significantly induced immunogenic cell death and increased tumor cell immunogenicity in vitro. Moreover, combined irradiation and lipotecan significantly upregulated cancer cell immunogenicity, compared to IR alone and lipotecan alone group.

### 3.3. Lipotecan Remarkably Enhanced the Therapeutic Efficacy of Radiotherapy In Vivo

**Figure 4:**
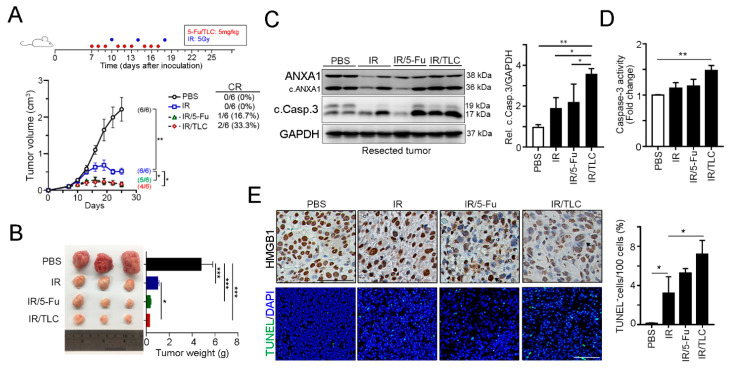
The TLC-based CRT regimen significantly suppressed tumor growth in vivo. (**A**) Tumor growth of CT26-driven colon carcinoma established in BALB/c mice (*n* = 6 per group) that were treated with IR (5 Gy for 3 fractions), IR/5-Fu (5-Fu: 5 mg/kg) and IR/TLC (TLC: 5 mg/kg). Tumor growth is reported as the mean tumor volume ± SEM. over time. * *p* < 0.05 and ** *p* < 0.01. CR: complete response. (**B**) The weight of resected tumors was examined (*n* = 3). * *p* < 0.05 and *** *p* < 0.001. (**C**) Resected tumors were extracted, and the protein levels were analyzed by immunoblotting (*n* = 3). (**D**) Caspase-3 activity was measured by caspase-3 activity assay (*n* = 3). ** *p* < 0.01. (**E**) The representative results of HMGB were analyzed by immunohistochemistry (*n* = 3), and the extent of cell death was measured by TUNEL assay (*n* = 3). * *p* < 0.05. More details of western blot, please view at Figure S5.

To verify the therapeutic efficacy and antitumor immunity of lipotecan in colorectal cancer, we inoculated BALB/c mice with CT26 mouse colon carcinoma cells and treated the mice with 5-Fu-based and lipotecan-based concurrent chemoradiotherapy (CRT) regimens (Figure 4A). As shown in Figure 4A, tumor growth was suppressed by radiotherapy. Either 5-Fu or lipotecan has a synergetic effect with radiotherapy to inhibit tumor growth. Although the average tumor inhibition was similar in the 5-Fu-based CRT and lipotecan-based regimen, we found that lipotecan-based CRT (IR/TLC) had a better clinically complete response (CR, 2/6 = 33.3%, Figure 4A) than the 5-Fu-based CRT regimen (IR/5-Fu, 1/6 = 16.7%, Figure 4A). Moreover, the weights of resected tumors from the 5-Fu-based and lipotecan-based CRT regimens were remarkably smaller than those of the PBS group (Figure 4B).

Furthermore, the immunoblotting results showed that phosphorylation of eIF2α and cleavage of caspase-3 were clearly observed in the resected tumors treated with the lipotecan-based CRT regimen (Figure 4C). The activity of caspase-3 was also increased in the IR/TLC group (Figure 4D). The results of immunohistochemical staining showed that HMGB1 was clearly released from the nucleus into the cytoplasm in the IR/TLC group (Figure 4E). Moreover, the results of the TUNEL assay revealed that lipotecan-based CRT also significantly induced cell death. Taken together, these results showed that the lipotecan-based CRT regimen directly damaged cancer cells and ICD in vivo.

### 3.4. Lipotecan-Based CRT Provoked Antitumor Immunity to Enhance Therapeutic Efficacy In Vivo

**Figure 5:**
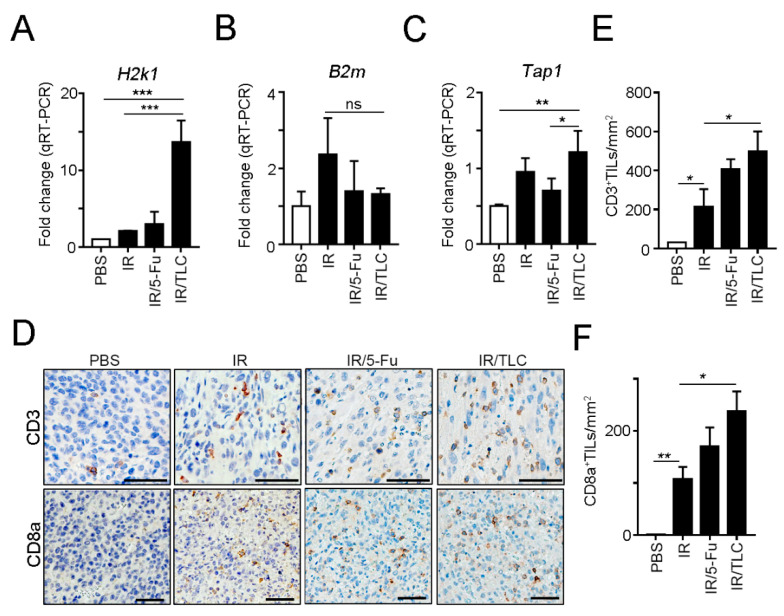
The TLC-based CRT regimen significantly provoked immunogenicity and recruitment of T cells in vivo. (**A**) The level of *H2k1* in resected tumors was analyzed by qRT-PCR (*n* = 3). *** *p* < 0.001. (**B**) The level of *B2m* in resected tumors was analyzed by qRT-PCR (*n* = 3). (**C**) The level of *Tap1* in resected tumors was analyzed by qRT-PCR (*n* = 3). * *p* < 0.05 and ** *p* < 0.01. (**D**) The representative results were infiltration of CD3^+^ TILs, and CD8a^+^ TILs were analyzed by immunohistochemistry (*n* = 3). (**E**) The density of CD3^+^ TILs was counted under high-power-field microscopy (*n* = 3). * *p* < 0.05. (**F**) The density of CD8a^+^ TILs was counted under high-power-field microscopy (*n* = 3). Quantitative analysis of the immunoblotting results. * *p* < 0.05 and ** *p* < 0.01.

To confirm that the lipotecan-based CRT regimen enhanced cancer immunogenicity and recruitment of T cells into the tumor microenvironment, we evaluated the levels of *H2k1*, *Tap1* (transporter associated with antigen processing 1), and *B2m* (β2-microglobulin, which is a component of the MHC class I molecule) by qRT-PCR (Figure 5A–C). These results showed that lipotecan-based CRT indeed increased *H2k1* and *Tap1* expression for cancer immunogenicity (Figure 5A,C) and had no influence on *B2m* expression (Figure 5B), which indicated that lipotecan remodulated cancer immunogenicity within the TME. Moreover, the recruitment of CD3^+^ TILs and cytotoxic CD8a^+^ TILs was remarkably increased (Figure 5D–F). The density of CD3^+^ TILs and CD8a^+^ TILs was significantly upregulated in the IR/TLC group. These results indicated that lipotecan-based CRT enhanced cancer immunogenicity to recruit T cells for antitumor immunity in vivo.

## 4. Discussion

In this study, we uncovered the potential therapeutic effect of lipotecan on remodeling cancer immunogenicity within the TME and promoting ICD for anticancer immunity to eradicate residual cancer cells. Low-dose lipotecan not only damages cancer cells but also triggers ER stress for CRT surface exposure and HMGB1 and ANXA1 release, regarded as the characteristics of ICD induction. Moreover, lipotecan remarkably with radiotherapy enhances TAP1 and MHC class I expression for antigen presentation, reinvigorating cancer immunogenicity for tumor-specific T cell activation and leading to favorable complete regression ability in vitro and in vivo. Taken together, these results indicate that the novel topoisomerase I inhibitor lipotecan fosters radiotherapy-induced anticancer immunity, providing new therapeutic strategies for improving the therapeutic efficacy of the neoCRT regimen.

The current standard preoperative neoCRT regimen for LARC patients achieves complete tumor regression in 15–20% of patients with lower locoregional recurrence. However, 30–40% of LARC patients only achieve partial response [40], suffer the unnecessary toxicity of drug effect, and have an increased risk of distant metastasis within five years. Therefore, several cytotoxic agents have been extensively explored in clinical trial settings to improve the therapeutic efficacy of neoCRT regimens, such as oxaliplatin [16] and topoisomerase inhibitor irinotecan [14]. Bains et al. recently reported that short-course oxaliplatin before neoCRT provokes a pronounced rise in soluble immune factor HMGB1 that remained elevated during sequential neoCRT [15]. Moreover, their results showed that patients who responded to neoCRT had significantly better progression-free survival than patients without such responses, indicating that an advantageous systemic immune response had been invoked by oxaliplatin to increase the therapeutic efficacy of the standard neoCRT regimen [15]. Kitai et al. indicated that the topoisomerase I inhibitor topotecan triggered the secretion of DAMPs to promote DC maturation and CD8^+^ T cell activation to delay tumor growth in vivo [19]. TPT treatment resulted in the secretion of exosomes containing immunostimulatory DNA from cancer cells, further activating DCs via a STING-dependent pathway [19]. Thus, these findings suggest that topoisomerase I inhibitors have the potential to act as immunostimulatory adjuvants to elicit antitumor immunity. Similarly, our findings indicate that topoisomerase I inhibitor induces ICD and increases cancer immunogenicity to remodel the TME. Our previous studies indicated that the release of HMGB1 and infiltration of CD8^+^ TILs were significantly associated with favorable survival outcomes in colorectal cancer [34,41,42], especially in patients who received the neoCRT regimen [34,41]. Consistent with our findings, Bains et al. showed that oxaliplatin-based neoCT-induced increases in circulating HMGB1 were positively correlated with better disease-free survival in high-risk LARC patients who received neoCRT, indicating that HMGB1 is regarded as a surrogate of ICD induction for anticancer immunity within the TME. Circulating HMGB1 promotes dendritic cell maturation and activation via Toll-like receptor 4 (TLR4), which facilitates the cross-presentation of shed tumor antigens by dendritic cells to activate tumor-specific cytotoxic T cells. Radiotherapy and a few DNA damage chemotherapeutic drugs, such as oxaliplatin (alkylating agent) and anthracycline (topoisomerase II inhibitor), are well-known cytotoxic agents that provoke these responses, which theoretically may unleash systemic antitumor effects that eradicate residual tumor cells. In the present study, we found that the novel DNA damage agent lipotecan remarkably releases HMGB1 and ANXA1 at low doses to prompt ICD and increase cancer immunogenicity in vitro and in vivo, suggesting that topoisomerase I (Top I) inhibitors may have the potential to elicit ICD and anticancer immunity. Supporting our observation, McKenzie et al. recently indicated that Top I inhibitors improved the antitumor efficacy of T cell-based cancer immunotherapy, implying that Top I inhibitors may unleash shed tumor antigens to increase cancer immunogenicity to augment the efficacy of immunotherapy [43]. Our results indicated that lipotecan upregulates TAP1 and MHC class I expression to facilitate tumor antigen presentation, thereby attracting cytotoxic T lymphocyte infiltration. Although there is no significant difference in tumor volume between the 5-Fu-based CRT and lipotecan-based CRT regimen in vivo, the complete response rate of the lipotecan-based regimen is better than the 5-Fu-based CRT regimen, suggesting that lipotecan might induce better anticancer immunity to eradicate the residual tumors, especially the infiltration of CD8^+^ immune cells. Supporting these results, Top I inhibitor topotecan remarkably promoted anticancer immunity in a STING-dependent manner [19]. We assume that lipotecan may also activate other mechanisms such as the STING-dependent pathway via dsDNA to increase the anticancer immune response of radiotherapy. Moreover, compared to the dosage of 5-Fu (50 mg/kg), low-dose lipotecan (5 mg/kg) combined with radiotherapy dramatically delayed tumor growth and recruited more cytotoxic T lymphocyte infiltration within TME, suggesting the minimal dose of lipotecan can elicit a similar therapeutic response of high-dose 5-Fu. Low-dose lipotecan not only minimizes the drug toxicity but induces the complete response rate, providing potential therapeutic strategies for increasing the therapeutic efficacy of neoCRT. Supporting our findings, Huang et al. demonstrated that lipotecan was a radiosensitizing agent in vitro and provides ~2.0-fold inhibition of topoisomerase I activity compared to topotecan [31]. Moreover, they found lipotecan increased the cell death ratio of radiotherapy ~2.0 in vitro by evaluating the sensitizer enhancement ratio (SER), which is the slope of cell death curve of lipotecan/IR versus the slope of the cell death curve of IR, suggesting lipotecan has the synergetic effect on radiotherapy. However, there is a lamination in our study. Our studies did not evaluate the radiosensitivity parameters α and β for the linear–quadratic (LQ) model to define the equivalent biological effective dose (BED) of lipotecan and radiotherapy in vitro and in vivo. Therefore, further studies are needed to evaluate the combined effect of lipotecan and radiotherapy to optimize the dosing and timing of lipotecan administration in the neoCRT regimen.

In summary, this study provides evidence that lipotecan has a potential immunologic effect to remodulate the TME by both inducing ICD and increasing cancer immunogenicity in vivo. When the optimization of the dosing and timing of lipotecan administration are known, lipotecan-based neoCRT may improve outcomes in the clinic.

## 5. Conclusions

These results show that the novel topoisomerase I inhibitor lipotecan is an immunologic chemotherapeutic agent that increases tumor immunogenicity and reinvigorates anticancer immunity, thereby prompting the therapeutic efficacy of radiotherapy in vivo. Therefore, lipotecan-based preoperative CRT may provide a novel therapeutic strategy to improve outcomes in CRC patients.

## Data Availability

The data that support the findings of this study are available from the corresponding author KS Clifford Chao upon reasonable request.

## Supplementary Materials

The following are available online at https://www.mdpi.com/2072-6694/13/6/1218/s1, Figure S1. Original western blot of Figure 1B, Figure S2. Original western blot of Figure 1C, Figure S3. Original western blot of Figure 2A, Figure S4. Original western blot of Figure 2B, Figure S5. Original western blot of Figure 4C.
