# Supplementary material for: Immunogenic Cell Death by the Novel Topoisomerase I Inhibitor TLC388 Enhances the Therapeutic Efficacy of Radiotherapy
Source: Cancers (Basel). 2021 Mar 11;13(6):1218. doi: 10.3390/cancers13061218 (PMC7998596; doi:10.3390/cancers13061218)
Supplement: Supplementary file 1 [file cancers-13-01218-s001.pdf]

cancers

Supplementary Materials

Immunogenic Cell Death by the Novel Topoisomerase I Inhibitor TLC388 Enhances the Therapeutic Efficacy of Radiotherapy

Kevin Chih-Yang Huang, Shu-Fen Chiang, Pei-Chen Yang, Tao-Wei Ke, Tsung-Wei Chen, Ching-Han Hu, Yi-Wen Huang, Hsin-Yu Chang, William Tzu-Liang Chen and K. S. Clifford Chao

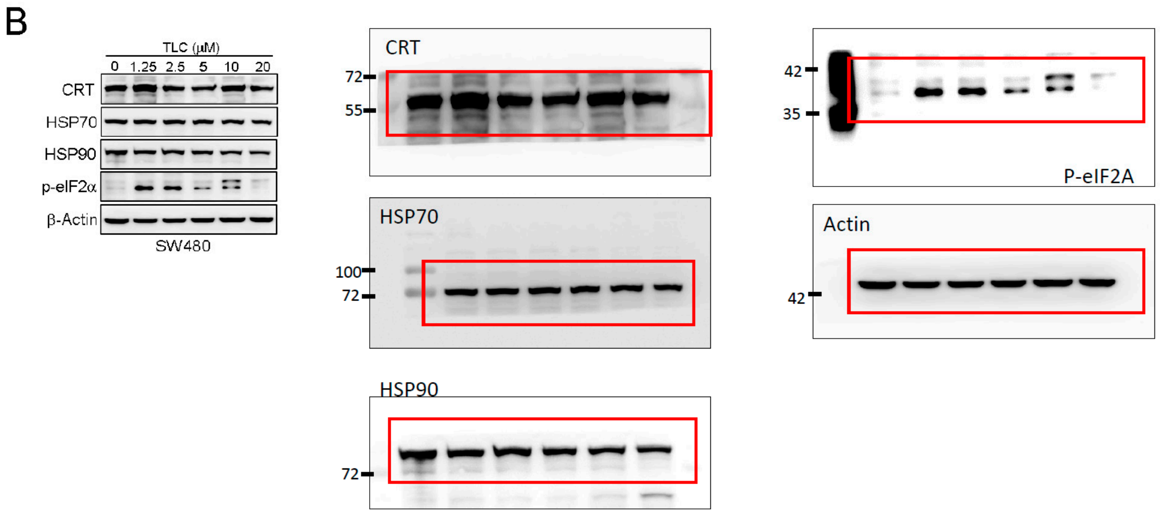

Figure 1B

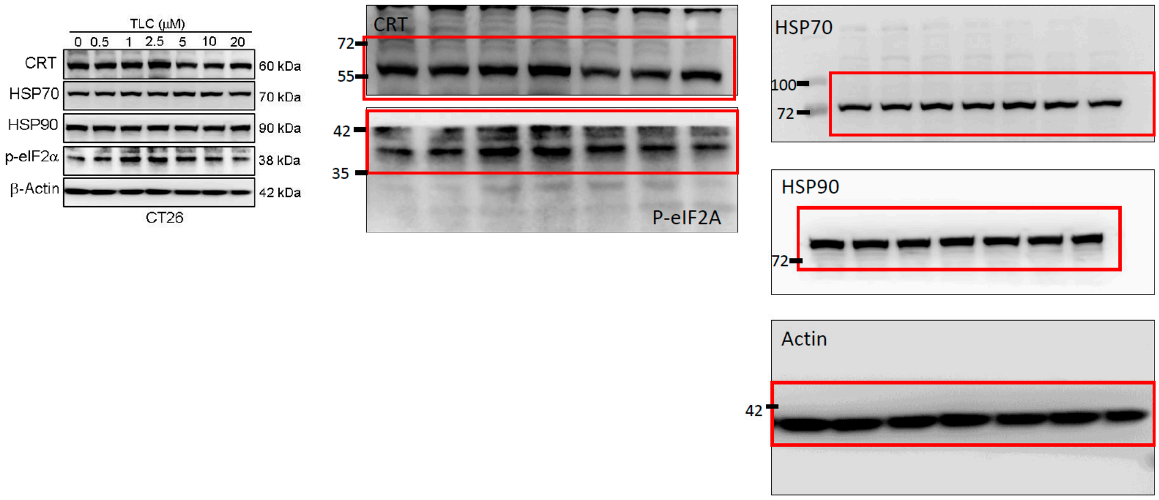

Figure S1. Original western blot of Figure 1B.

Figure 1C

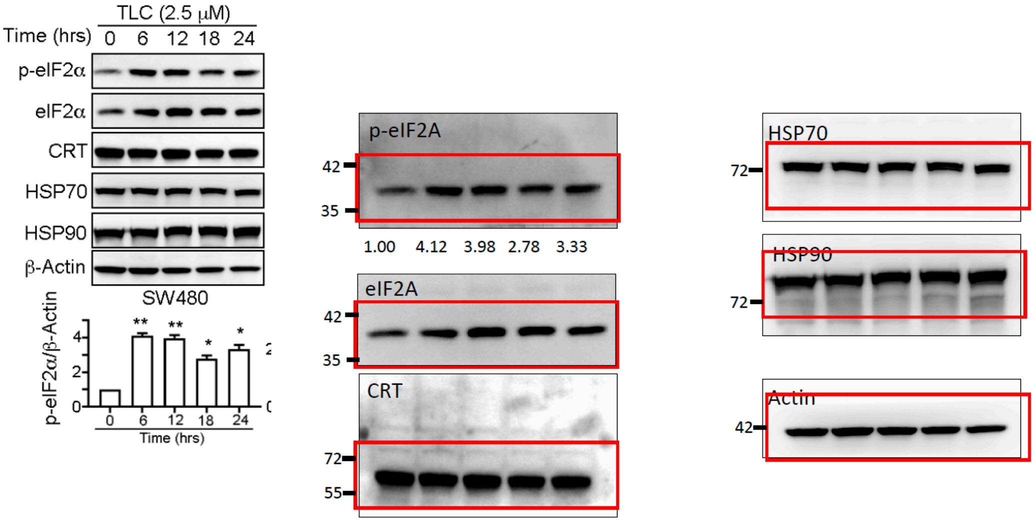

Figure 1C

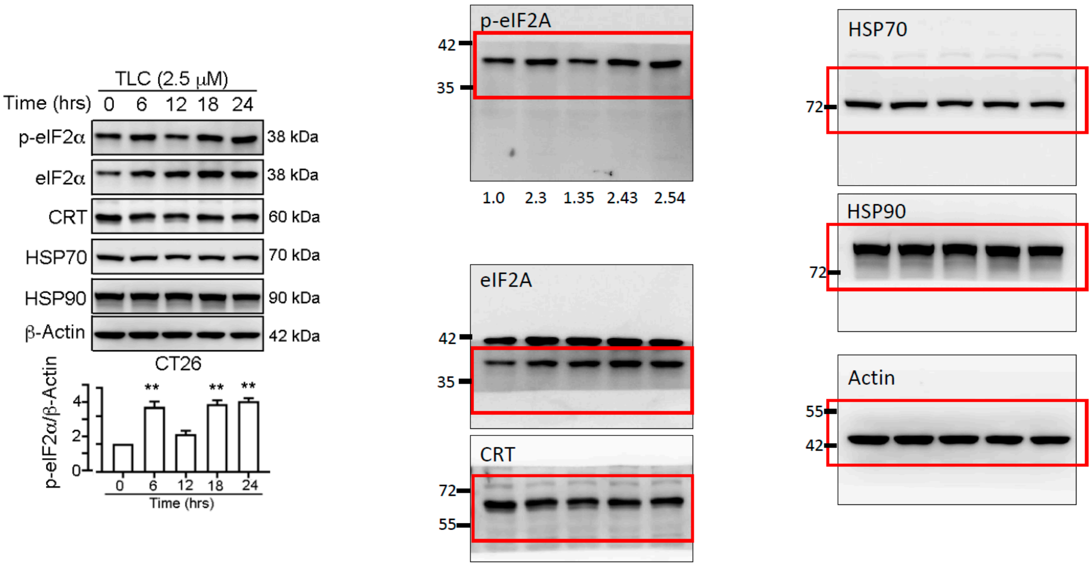

Figure S2. Original western blot of Figure 1C.

Figure 2A

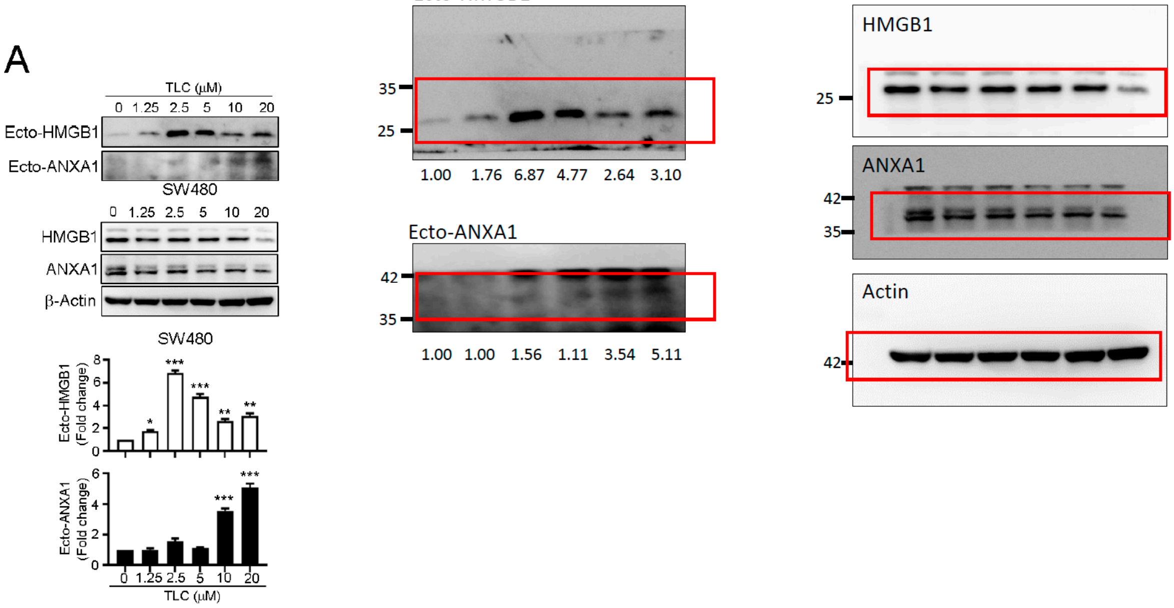

Figure 2A

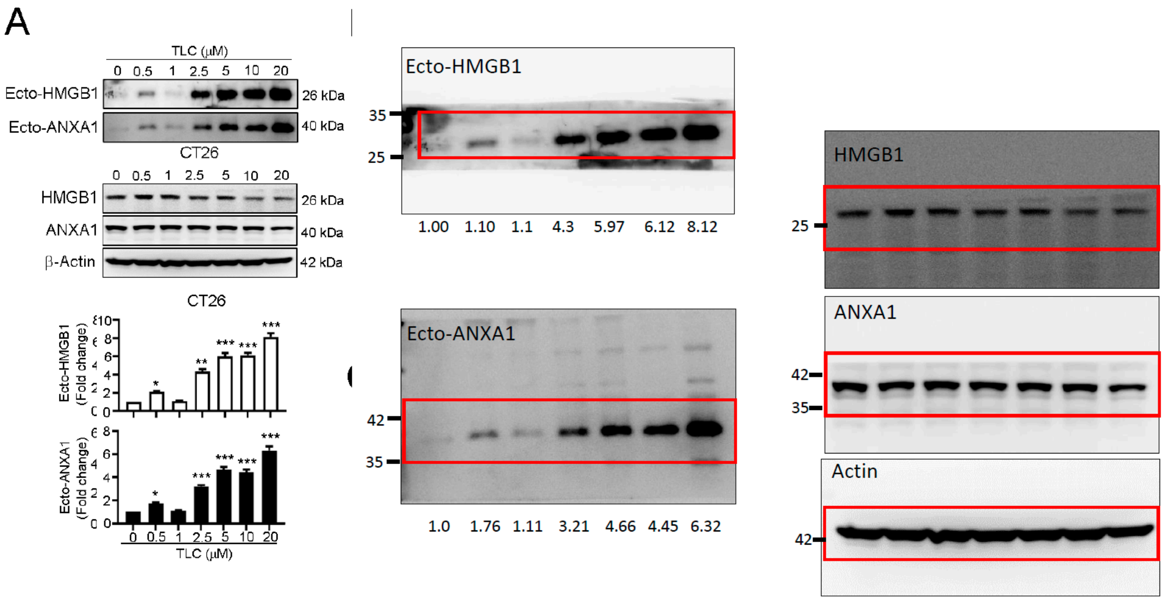

Figure S3. Original western blot of Figure 2A.

Figure 2B

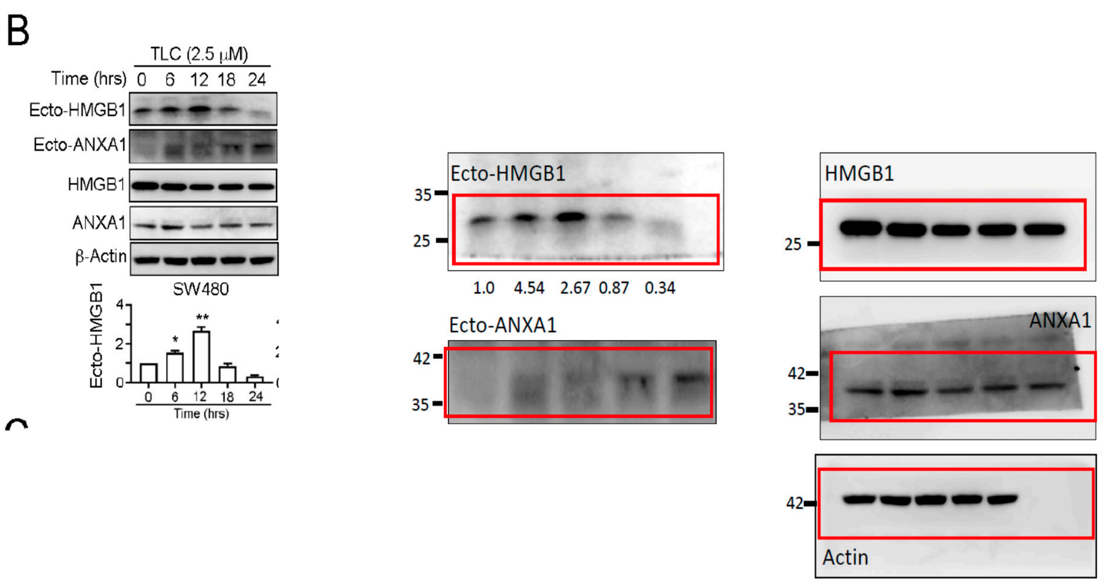

Figure 2B

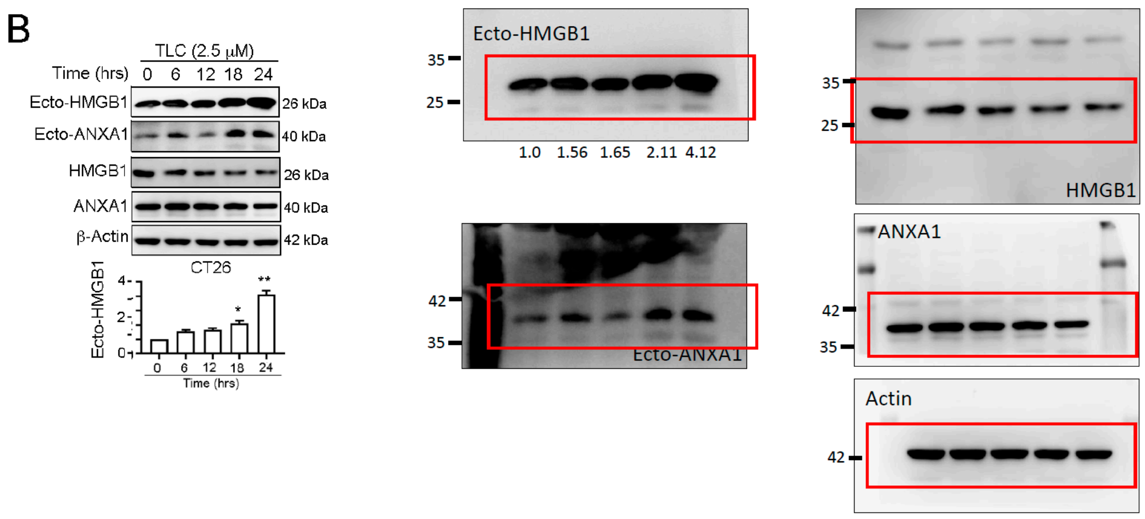

Figure S4. Original western blot of Figure 2B.

Figure 4C

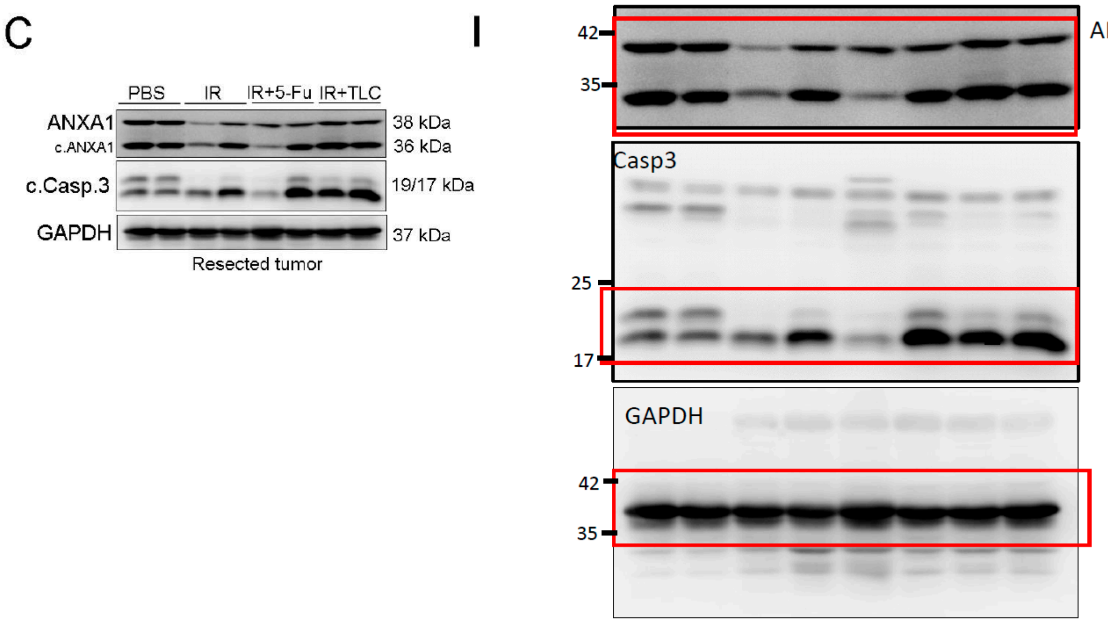

Figure S5. Original western blot of Figure 4C.
